# Chronological Progression of an Enlarged Styloid Process: A Case Report of Eagle Syndrome

**DOI:** 10.1155/2018/9207264

**Published:** 2018-01-15

**Authors:** Daisuke Maki, Kenji Okami, Koji Ebisumoto, Akihiro Sakai

**Affiliations:** Department of Otolaryngology, Head and Neck Surgery, Tokai University, Kanagawa, Japan

## Abstract

Eagle syndrome is characterized by an elongated styloid process. However, the time frame over which the styloid process becomes elongated and extends is unknown. How the condition worsens over time is also unclear. To date, there has been no report describing the chronologic change that occurs in the styloid process in Eagle syndrome. We describe a 53-year-old patient with Eagle syndrome in whom the styloid process enlarged progressively over time and the elongated styloid process fused with the hyoid bone. The styloid process was resected via a transcervical approach, and the patient's subjective symptoms improved. This is the first report showing how the styloid process can enlarge over a few years in a patient with Eagle syndrome. Surgical resection via a transcervical approach is an appropriate treatment for a patient in whom the styloid process has become excessively enlarged and elongated.

## 1. Introduction

Eagle syndrome (an elongated styloid process) causes a range of symptoms because of physical irritation by mastication and swallowing and nerve compression by the deformed styloid process [1]. Although many case reports have been published, complete connection of the styloid process to the hyoid bone is rare, and no study has described the natural history of the enlarging course of the elongated styloid process. Herein, we report the case of a patient with a massively enlarged styloid process in whom sequential changes were observed by chance.

## 2. Case Presentation

**Figure 1:**
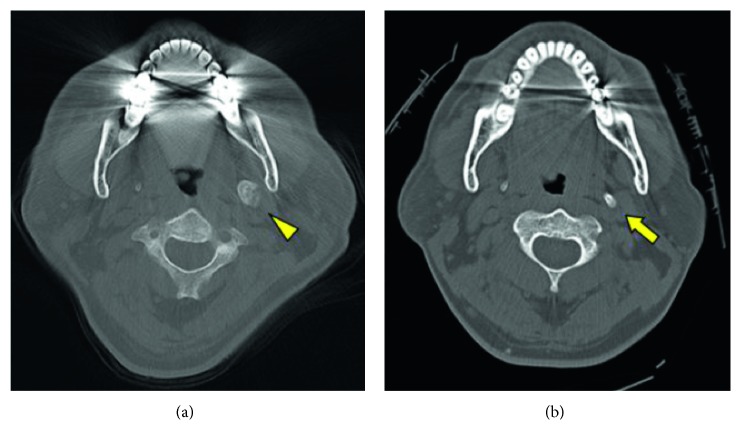
(a) Preoperative computed tomographic image showing enlargement of the left styloid process to approximately 10 mm thick (arrowhead). (a) A computed tomographic image taken 5 years earlier showing enlargement of the styloid process to around 5–7 mm thick (arrow) when the patient was examined for a facial injury.

**Figure 2:**
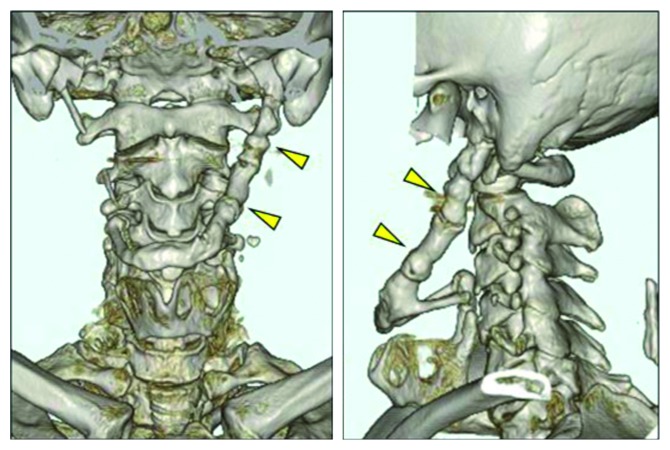
Three-dimensional reconstruction of a computed tomographic image taken preoperatively showing that the left styloid process was fused completely to the hyoid bone and had a total length of 70 mm (arrowheads). It was approximately 10 mm thick along almost its entire length.

A 53-year-old man presented to our institution with a 3-year history of pain in the left throat and trismus caused by pain. Magnetic resonance imaging in the Department of Neurology revealed an abnormal shadow above the hyoid bone. The patient was referred to the Department of Otorhinolaryngology, where no abnormal signs other than trismus were found. Computed tomography (CT) revealed that the left styloid process was connected to the hyoid bone, had a total length of 70 mm, and was approximately 10 mm thick along almost its entire length (Figures 1 and 2). Five years earlier, the patient had been transferred to our emergency clinic with a facial injury, and cervical CT images taken also revealed elongation of the styloid process. At that time, the styloid process had been approximately 5–7 mm thick, and its enlargement in the subsequent 5 years was evident (Figure 1).

**Figure 3:**
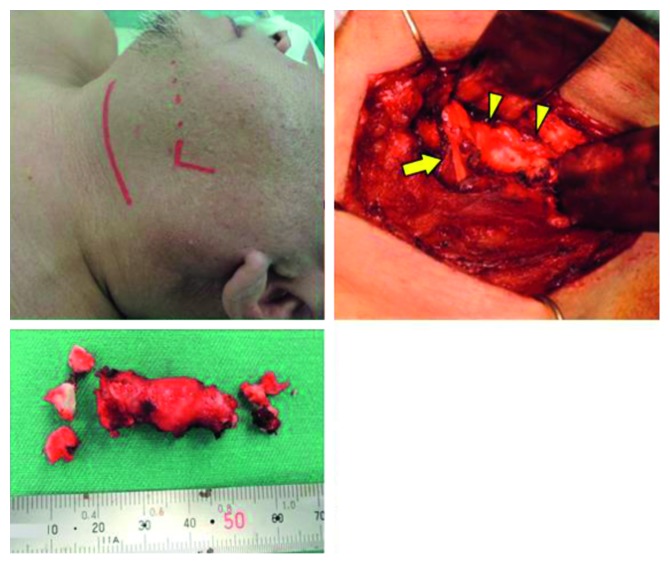
Intraoperative findings and the resected styloid process. The styloid process was resected via a transcervical approach. The submandibular gland was removed to ensure a clear field of view. The styloid process (arrowheads) was observed to be continuous with the hyoid bone on the dorsal side of the hypoglossal nerve (arrow), and a 40 mm portion was resected from the site of attachment to the hyoid bone.

**Figure 4:**
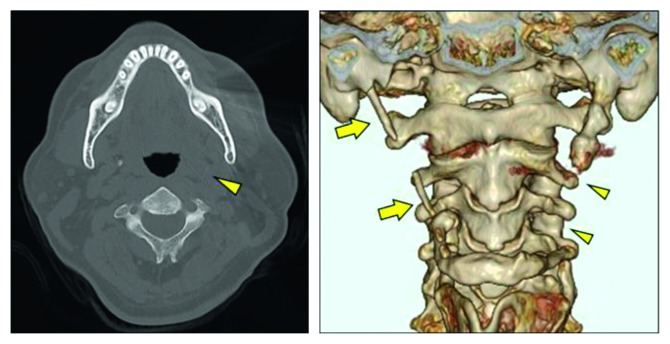
Postoperative computed tomographic image and three-dimensional reconstruction. A good portion of the left styloid process was resected (arrowheads). The styloid process on the right side was also elongated, and the stylohyoid ligament was partially calcified (arrows). Ongoing observation is required.

The patient was diagnosed with Eagle syndrome and treated initially with analgesics. However, the treatment with analgesics had not improved his pain, so he underwent transcervical resection of the styloid process under general anesthesia. A horizontal skin incision was made in the upper part of the neck, and the submandibular gland was removed to ensure a wide surgical field (Figure 3). The styloid process was firmly connected to the hyoid bone on the dorsal side of the hypoglossal nerve, and a 40 mm portion was resected from the attachment to the hyoid bone. No invasion of the surrounding tissue was found intraoperatively, and it was easy to separate and resect the bone. Pathologic examination revealed that the lesion consisted of bony tissue with a bone marrow component and a cartilaginous component in contact with the trabecular bone. There were no signs of active inflammation or malignancy. The symptoms such as pain and trismus disappeared completely. Preoperatively, the patient scored pain on opening his mouth and pain on swallowing as 8 on a visual analog scale, and both scores improved to 0 postoperatively. The patient had no recurrence of subjective symptoms at the most recent follow-up 19 months postoperatively. Postoperative CT revealed that a good portion of the left styloid process was resected (Figure 4). The stylohyoid ligament on the right side was partially calcified and requires ongoing observation in the future.

## 3. Discussion

Eagle syndrome causes a wide range of symptoms because of elongation of the styloid process and calcification of the stylohyoid ligament [1]. Some of these symptoms are attributable to direct physical irritation, while others are caused by compression of branches of the glossopharyngeal and vagal nerves. Symptoms include laryngopharyngeal pain, pain on swallowing, dysphagia, earache, trismus, pain when turning the neck, and facial pain. Eagle syndrome may also cause internal carotid artery dissection [2, 3]. Although the cause of Eagle syndrome is unknown, abnormal bone metabolism and developmental anomalies are thought to be involved [4]. The styloid process is normally 25–30 mm in length, but according to Eagle et al., the styloid process measures ≥25 mm in approximately 4% of adults, and approximately 4% of these individuals will exhibit symptoms [1, 2]. Patients with this syndrome may be treated conservatively or surgically. No study has demonstrated the long-term efficacy of conservative therapy, such as oral analgesics or a nerve blocking agent. Surgery is recommended as a curative treatment and may be performed via a transoral approach or a transcervical approach [5]. The oral approach involves first removing the palatine tonsil and making an incision in the pharyngeal constrictor muscle to resect the hyoid process lateral to it. This approach has the advantages of a shorter operating time, a shorter period of hospitalization, and not requiring a skin incision. The transcervical approach involves resecting the hyoid process from below the mandible via a skin incision in the neck. This approach provides a broad field of view ensuring clear visibility of the anatomic structures and enabling resection of a large portion of the hyoid process; however, it has the disadvantages of a longer period of hospitalization, a longer operating time, and leaving a scar on the neck [6]. Although there have been almost no studies comparing the postoperative courses after these two procedures, a comparative report of a small number of patients found no major difference in visual analog scale scores for subjective symptoms [5].

Patients such as the one reported here in whom the styloid process was thick and completely continuous with the hyoid bone are rare, with only three previous cases reported [7–9]. The patient's course, including treatment, was unknown in one patient, but surgery was performed via the external cervical approach in the other two patients. This procedure is recommended for patients with a long and large styloid process because the oral approach does not provide an adequate field of view.

To the best of our knowledge, no previous case report of Eagle syndrome has described the enlarging course of the elongated styloid process. The present case was unusual in that the styloid process could be seen to have approximately doubled in thickness over a 5-year period. The styloid process does not usually enlarge over time; however, the contralateral styloid process extended in this case and the stylohyoid ligament became calcified. Therefore, it was thought that this patient had some unusual features. He had been punched in the face 5 years earlier and suffered a traumatic cervical syndrome. There is a report suggesting that inflammation can cause elongation of the styloid process [10]. The injury-related inflammation might have caused the elongation in this case. Although the patient consented to surgery only on the left side, careful follow-up of the right side is needed.

Although the etiology of the development of Eagle syndrome has been considered for a long time, the exact cause is not known. As in our present case, a styloid process may become excessively elongated. If elongation of the styloid process is discovered by chance, it should be explained to the patient that the elongation may worsen and that subjective symptoms may occur.
